# The Doctor

**Published:** 1896-11

**Authors:** 


**The Doctor.**—Of all lives the life of the physician is the most self-denying. He has no time that he can call his own. His home is his office, and furnishes him no sweet retreat from irksome care. The night can never assure him unbroken rest. Sundays are often, whether he will or no, his busiest days. He has no holidays, and few and fragmentary vacations. Friendship furnishes him fewer solaces than to other men, for his friends are generally also his patients. He meets men in their morbid conditions—when they are sick and miserable; when they are well he knows them not. He can hardly make a friendly call without the hazzard of having it converted, before the evening is over, into a professional one. He fights a battle in which, no matter how many victories he wins, he is sure to be defeated at last—for he is fighting death. And when the defeat, which must come sooner or later, does come, he is fortunate if unreasonable friends do not charge the defeat upon his lack of science or of care. But no man renders a more grateful service; no man comes nearer to our hearts; no man is more beloved. Other services may be as great, but none is more deeply and tenderly appreciated. He summons back from death the child, and puts him in his mother's arms; the wife, and reunites her to her husband. No fee can ever compensate for such a service. He to whom it is rendered is forever debtor to the doctor.—*The Outlook.*

---

**A Brooklyn** physician, translating from a German writer, thus discourses on apples as food and medicines: "The apple is such a common fruit that few person are familiar with its remarkably efficacious medicinal properties. Everybody ought to know that the very best thing he can do is to eat apples just before going to bed. The apple is excellent brain food because it has more phosphoric acid, in an easily digestible shape than any other fruit known. It excites the action of the liver, promotes sound and healthy sleep, and thoroughly disinfects the mouth. It also agglutinates the surplus acids of the stomach, helps the kidney secretions and prevents calculus growth, while it obviates indigestion and is one of the best preventives of diseases of the throat. Next to lemon and orange it is also the best antidote for the thirst and craving of persons addicted to the alcohol and opium habit."—*Wm. Kinnear, The North American Review.*

---

**Johnny** was to make his initial appearance in public at the Sunday-school concert. His mother selected for him to repeat the words, "I am the light of the world," and when the evening of the concert arrived, Johny came forward and proclaimed in a loud voice, "My mother is the light of the world." "So she is of your little world, laddie," said the superintendent, as a smile of satisfaction beamed on many faces. Johnny had not made so great a mistake after all.—*School Physiological Journal.*

---

**Racking** headaches can often be relieved magically, as any lover of the luxury of a graceful barber and a comfortable barber's chair can testify, by the application of towels wrung out in boiling water and laid across the forehead, eyes and part of the head which seems to be most affected.—*I. N. Love.*

---

**Dr. O. W. Bramer** treated thirty cases of diphtheria with tincture chloride of iron internally, and local sprays of creolin without a single death. Of three cases, no worse than some of the others, treated with antitoxine, one died; a mortality of $33\frac{1}{3}$ per cent.—*Journal American Medical Association.*

